# Association between tear lactoferrin, lysozyme, albumin levels, and obstructive sleep apnea syndrome: a cross-sectional study

**DOI:** 10.3389/fmed.2025.1584473

**Published:** 2025-06-06

**Authors:** Bin Wang, Chaoyang Hong, Wenwei Li

**Affiliations:** ^1^The Second Clinical Medical College, Zhejiang Chinese Medical University, Hangzhou, Zhejiang, China; ^2^Department of Ophthalmology, Tongde Hospital of Zhejiang Province, Hangzhou, Zhejiang, China; ^3^Department of Ophthalmology, Zhejiang Provincial People's Hospital, Hangzhou, Zhejiang, China

**Keywords:** obstructive sleep apnea syndrome, dry eye, tear, lactoferrin, lysozyme, albumin

## Abstract

**Background:**

Obstructive Sleep Apnea Syndrome (OSAS) is associated with various ocular diseases, but its relationship with tear biomarkers for dry eye remains unclear. This study aimed to investigate these tear biomarkers and their correlation with OSAS.

**Methods:**

This cross-sectional study was conducted at a tertiary hospital in Zhejiang Province from January 2021 to December 2022. Forty-one OSAS patients were enrolled. Tear lactoferrin, lysozyme, and albumin levels (exposure variables) were measured using ELISA, while OSAS (outcome variable) was diagnosed through polysomnography. Age, gender, and BMI were collected as covariates.

**Results:**

Baseline characteristics (including age, gender, and BMI) showed no statistical differences between groups. No significant correlations were found between tear albumin (*r* = 0.10, *p* > 0.05), lactoferrin (*r* = 0.18, p > 0.05), lysozyme (r = −0.05, p > 0.05) levels and OSAS.

**Conclusion:**

This study found no significant correlation between dry eye tear biomarkers levels and disease severity in OSAS patients. Larger-scale studies are needed to validate these findings.

## 1 Introduction

Obstructive Sleep Apnea Syndrome (OSAS) is a common sleep disorder characterized by recurrent upper airway collapse during sleep, leading to intermittent hypoxia and sleep fragmentation (1). OSAS affects approximately 2–4% of the adult population worldwide, significantly impacting patients’ quality of life and closely associated with various cardiovascular and metabolic diseases (2–4).

Recent studies have shown that OSAS is associated with several ocular diseases, such as dry eye syndrome, glaucoma and retinopathy (5–7). Tear fluid is an essential component in maintaining ocular surface health, and abnormalities in its composition and function may be an important underlying cause of OSAS-related ocular complications (8–10). In recent years, there have been many studies on biomarkers of dry eye and other ocular surface diseases (11–15). Previous studies have reported significantly elevated levels of certain inflammatory factors, such as interleukins (IL) and tumor necrosis factor (TNF), in the tear fluid of OSAS patients (16–18). Tear lactoferrin, lysozyme and albumin are considered to be the most common biomarkers for dry eye disease in recent years (19–29).

However, current research on the changes in tear biomarkers for dry eye in OSAS patients and their correlation with OSAS severity is limited, and the underlying mechanisms remain unclear. Given the high prevalence of OSAS and its widespread impact on overall health, understanding the relationship between OSAS and tear biomarkers for dry eye has significant scientific and clinical value. Therefore, this study aims to detect the levels of major biomarkers for dry eye in the tear fluid of OSAS patients and analyze their correlation with OSAS severity, with the goal of providing new insights into the pathogenesis of OSAS-related ocular complications and offering potential clinical implications for diagnosis and treatment.

## 2 Materials and methods

### 2.1 Study population

This is a cross-sectional study conducted from January 2021 to December 2022 at a tertiary hospital in Zhejiang Province. The study population consists of 80 patients diagnosed with sleep apnea syndrome. The inclusion criteria are: age ≥18 years, meeting the diagnostic criteria for OSAS, no history of any surgery or drug treatment, and no history of severe diseases such as cardiovascular, hepatic, pulmonary, renal, hypertension, diabetes, or tumors, as well as no history of eye or mental disorders. The exclusion criteria are: age <18 years, history of eye diseases or surgery, history of other surgeries or long-term drug treatment, history of the above-mentioned severe diseases, history of mental illness, and inability or unwillingness to participate in the study. All data were obtained from the hospital’s electronic medical record system.

### 2.2 Sample collection

Tear samples were collected within 24 h after completion of PSG testing. Tear fluid was collected under controlled ambient conditions (dim lighting, no topical anesthesia) to minimize external stimulation. Using sterile 50 μL glass capillary tubes (single-use per subject), tear samples were gently drawn from the inferior conjunctival cul-de-sac via passive capillary action, ensuring a minimum volume of 15 μL per collection. Care was taken to avoid mechanical irritation or reflex tearing during the procedure. Immediately after collection, tears were transferred to disposable 1.5 mL Eppendorf tubes. All samples were rapidly frozen at −80°C within 60 min of collection and preserved without prior centrifugation until subsequent biochemical analysis.

### 2.3 ELISA

Concentrations of albumin (Alb), lysozyme (LZM), and lactoferrin (LTF) in tear fluid were analyzed using validated sandwich enzyme-linked immunosorbent assay (ELISA) kits. Albumin levels were determined with a Human Albumin ELISA Kit (EnzymeLink Biotech, Cat. No. ml062456; detection range: 5–80 mg/mL), lysozyme with a Human Lysozyme ELISA Kit (EnzymeLink Biotech, Cat. No. ml063028; 50–800 μg/mL), and lactoferrin with a Human Lactoferrin ELISA Kit (Elabscience, Cat. No. E-EL-H5200c; 250–4,000 μg/mL). Undiluted tear samples (10 μL pooled from both eyes) were diluted in kit-specific buffers (Alb: 1:20 in PBS with 1% BSA; LZM: 1:10 in calibrator buffer; LTF: 1:50 in assay matrix) to align with dynamic ranges. Diluted samples (100 μL) were loaded into antibody-coated wells and incubated for 2 h at 37°C in a humidity-controlled chamber (Shanghai Xinmiao, DNP-9082BS-3). After five washes with 0.05% PBS-Tween 20, horseradish peroxidase (HRP)-conjugated detection antibodies were added and incubated for 1 h at 25°C. Color development was initiated with 3,3′,5,5′-tetramethylbenzidine (TMB) substrate (10 min, dark), followed by reaction termination using 1 M H2SO4. Absorbance was measured at 450 nm (reference 570 nm for LTF) on a Thermo Scientific Multiskan microplate reader (+24VDC/5A). Standard curves (0–80 mg/mL Alb, 0–800 μg/mL LZM, 0–4,000 μg/mL LTF) exhibited linearity (R2 > 0.99), with intra-and inter-assay coefficients of variation <8%. Samples exceeding detection limits were reanalyzed (maximum 1:100 dilution) or excluded (Alb <5 mg/mL; LZM < 50 μg/mL; LTF < 250 μg/mL). Assay specificity was confirmed by <1% cross-reactivity with homologous proteins (e.g., transferrin in LTF assays), and negative/positive controls were included in all runs.

### 2.4 Variables

The exposure variables include lactoferrin, lysozyme, and tear fluid albumin in tear fluid, which were measured using ELISA. The outcome variable in this study is sleep apnea syndrome, which was diagnosed using polysomnography. The diagnosis of Obstructive Sleep Apnea Syndrome (OSAS) is based on an Apnea-Hypopnea Index (AHI) of ≥5 events per hour, accompanied by clinical symptoms such as daytime sleepiness. OSAS can be staged as mild (5 ≤ AHI < 15), moderate (15 ≤ AHI < 30), or severe (AHI ≥ 30) based on the AHI value. The covariates included age, gender, BMI. The exposure and outcome variables were measured at the time of admission, and the assessment was conducted in a blinded manner.

### 2.5 Ethic statement

This study has been approved by the ethics committee of Tongde Hospital of Zhejiang Province. As this is a cross-sectional study, all participants have signed informed consent forms to agree to participate in the research. The study strictly adheres to the ethical principles of the Declaration of Helsinki to protect the rights of the participants.

### 2.6 Statistics analysis

Continuous variables (age, BMI, albumin, lysozyme, and lactoferrin levels) were compared among groups using one-way analysis of variance (ANOVA). Categorical variables (gender) were analyzed using chi-square test. Univariate analysis was performed to evaluate associations between potential risk factors and OSAS. Correlations between inflammatory factors and group classifications were assessed using correlation analysis with correlation coefficients (r). Data are presented as mean ± standard deviation (SD) for continuous variables and percentages for categorical variables. All statistical analyses were performed using R software (version 4.2.0, R Foundation for Statistical Computing, Vienna, Austria). A two-sided with *p* < 0.05 was considered statistically significant.

## 3 Results

A total of 41 participants met the inclusion criteria and were enrolled in this study. Participants were allocated into four groups: Group A (normal group, *n* = 11) and Groups B (mild OSAS), C (moderate OSAS), and D (severe OSAS) (*n* = 10 each for OSAS group). Baseline demographic and clinical characteristics were systematically analyzed to ensure between-group comparability (Table 1).

**Table 1:** Characteristics of study participants by groups of different degree of OSAS.

	Control group	Mild OSAS	Moderate OSAS	Severe OSAS	*p*-value
	*N* = 11	*N* = 10	*N* = 10	*N* = 10	
Age (years)	39.5 (12.2)	52.6 (16.0)	48.2 (14.8)	46.9 (12.3)	0.197
BMI (kg/m^2^)	29.3 (1.99)	28.9 (3.45)	27.4 (3.69)	27.4 (4.19)	0.447
Sex					0.618
F	6 (54.5%)	6 (60.0%)	3 (30.0%)	5 (50.0%)	
M	5 (45.5%)	4 (40.0%)	7 (70.0%)	5 (50.0%)	
Albumin (mg/mL)	57.8 (19.1)	54.2 (10.7)	59.1 (9.57)	60.2 (14.2)	0.795
Lysozyme (ng/mL)	443 (104)	443 (90.6)	445 (72.4)	432 (37.9)	0.981
Lactoferrin (ng/ml)	42.8 (50.2)	29.3 (44.2)	57.3 (52.8)	60.9 (54.3)	0.489

Although Group B demonstrated a nominally higher mean age (52.6 ± 16.0 years) compared to Groups A (39.5 ± 12.2 years), C (48.2 ± 14.8 years), and D (46.9 ± 12.3 years), this difference did not reach statistical significance (*p* = 0.197). It also revealed comparable body mass index (BMI) distributions across all groups (Group A: 29.3 ± 1.99 kg/m^2^, Group B: 28.9 ± 3.45 kg/m^2^, Group C: 27.4 ± 3.69 kg/m^2^, Group D: 27.4 ± 4.19 kg/m^2^; *p* = 0.447). With respect to gender distribution, female participants comprised 54.5, 60.0, 30.0, and 50.0% of Groups A, B, C, and D, respectively (χ^2^ test, *p* = 0.618). These findings collectively demonstrate that all measured baseline demographic and clinical parameters were comparable among the four study groups, thereby minimizing potential confounding effects in subsequent analyses.

Analysis of clinical parameters demonstrated consistent values across groups. The albumin measurements ranged from 54.2 ± 10.7 to 60.2 ± 14.2 mg/mL (*p* = 0.795), while lysozyme values remained stable across groups [Group A: 443 ± 104, Group B: 443 ± 90.6, Group C: 445 ± 72.4, Group D: 432 ± 37.9; (ng/mL); *p* = 0.981]. Similarly, lactoferrin measurements, despite showing wider variations (ranging from 29.3 ± 44.2 to 60.9 ± 54.3 ng/mL), exhibited no statistically significant differences between groups (*p* = 0.489).

As presented in Table 2, univariate analyses were conducted to evaluate the association between risk factors and OSAS. The analysis indicated that age, sex, BMI, albumin, lysozyme, and lactoferrin did not show a significant association with OSAS (*p* > 0.05).

**Table 2:** The results of univariate analysis.

Variables	Total (*n* = 41)	Group A (*n* = 11)	Group B (*n* = 10)	Group C (*n* = 10)	Group D (*n* = 10)	*p*	Statistic
Albumin, Median (Q1, Q3)	54.01 (49.85, 62.33)	51.44 (49.97, 56.45)	51.33 (49.85, 56.53)	58.5 (55, 60.6)	58.97 (49.14, 65.89)	0.518	2.273
Lysozyme, Median (Q1, Q3)	438.54 (389.08, 465.02)	413.23 (392.12, 441.8)	416.78 (384.69, 463.34)	454.97 (411.74, 485.76)	441.8 (408.78, 456.63)	0.752	1.203
Lactoferrin, Median (Q1, Q3)	17.74 (5.18, 104.9)	23.19 (5.01, 66.39)	7.79 (5.55, 25.25)	50.62 (8.14, 107.13)	59.42 (4.91, 116.01)	0.712	1.373
Year, Mean ± SD	46.61 ± 14.21	39.45 ± 12.23	52.6 ± 15.98	48.2 ± 14.8	46.9 ± 12.3	0.197	1.641
Sex, n (%)						0.618	Fisher
F	20 (48.78)	6 (54.55)	6 (60)	3 (30)	5 (50)		
M	21 (51.22)	5 (45.45)	4 (40)	7 (70)	5 (50)		
BMI, Median (Q1, Q3)	29.4 (26.7, 30.5)	29.5 (27.45, 30.9)	29.45 (27.05, 30.97)	27.65 (24.3, 29.7)	29.85 (23.05, 30.2)	0.647	1.657

Correlation analysis revealed varying degrees of associations between different parameters and group classification. A weak positive correlation was observed between albumin and different groups (*r* = 0.10, *p* > 0.05). Similarly, lactoferrin demonstrated a weak to moderate positive correlation with different groups (*r* = 0.18, *p* > 0.05). However, lysozyme showed negligible correlation with different groups (*r* = −0.05, *p* > 0.05). (Table 3) These findings suggest that while there are subtle relationships between these parameters and group classification, none reached statistical significance.

**Table 3:** Correlation between albumin, lysozyme, lactoferrin, and OSAS.

Variables	*p-*value	*r*
Albumin	>0.05	0.10
Lactoferrin	>0.05	0.18
Lysozyme	>0.05	−0.05

## 4 Discussion

This cross-sectional study enrolled 41 patients with Obstructive Sleep Apnea Syndrome (OSAS) to investigate the association between tear fluid inflammatory factors and disease severity. Using enzyme-linked immunosorbent assay (ELISA), the study measured lactoferrin, lysozyme, and albumin in tear fluid (exposure variables) and diagnosed OSAS severity through polysomnography (outcome variable). Results revealed no statistically significant correlations between these biomarkers and OSAS grouping (correlation coefficients r ranging from 0.05 to 0.19, all *p* > 0.05).

In our study, we found that biomarkers in tear fluid, such as lactoferrin, lysozyme, and albumin, did not show a significant correlation with the severity of Obstructive Sleep Apnea Syndrome (OSAS). This finding aligns with the results of Mackenzie et al., who conducted a study on the tear film in patients with OSAS and found no significant changes in inflammatory markers (matrix metalloproteinases, MMP-9) in 32 OSAS cases (30). Their study utilized similar methodologies, including the use of polysomnography for OSAS diagnosis and tear fluid analysis.

However, our results differ from those reported by Li et al. (2021), who did a metanalysis on 25 eligible studies, including 2,301 participants and 1,123 controls and they found that children and adults with OSAS have significantly elevated serum IL-8 concentrations (*p* < 0.001) (31). Ifergane made an analysis on 43 individuals experiencing acute stroke and sleep apnea, and they found that OSAS was associated with significantly increased serum levels of IL-6 (32). Wu included 100 participants, comprising 63 individuals with normal to moderate OSAS and 37 with severe OSAS, and they found that there was a significant interaction effect on serum IL-6 levels for all OSAS severity (*p* = 0.030) (33). Their study employed serum inflammatory markers, which might explain the observed differences with ours. The lack of significant OSA severity-tear biomarker associations contrasts with well-established systemic inflammation in OSAS. This discrepancy may arise from compartmentalized immune regulation: tear components serve constitutive barrier functions, whereas serum cytokines reflect acute-phase systemic inflammation. Additionally, tear sampling post-sleep may capture recovery-phase homeostasis, diluting transient nocturnal fluctuations. Future studies should integrate real-time tear collection during polysomnography to resolve temporal dynamics. The variation in findings could also be attributed to differences in study populations, the severity of OSAS among participants, and the specific inflammatory markers analyzed.

In conclusion, while our study did not find a significant correlation between tear biomarkers and OSAS severity, the differences observed in the literature highlight the complexity of OSAS-related ocular changes. Further research with larger sample sizes and comprehensive biomarker panels is needed to elucidate these relationships fully.

This study provides a novel perspective on understanding ocular complications associated with OSAS by systematically evaluating biomarkers for dry eye in tear fluid. Although no significant correlation was found between these markers and OSAS severity, this finding itself holds important clinical value and helps challenge and refine existing theoretical assumptions about OSAS-related ocular complications. The study’s unique contribution lies in its comprehensive assessment of multiple factors (including lactoferrin, lysozyme, and albumin), laying the groundwork for more precise future research. From a clinical practice perspective, the results suggest that clinicians should not overly rely on single markers when evaluating ocular health in OSAS patients, but instead adopt a more comprehensive and integrated assessment approach. Future research could expand sample sizes, incorporate more covariates, and utilize advanced molecular biological techniques to further explore the complex mechanisms underlying OSAS and ocular inflammation, ultimately providing more valuable scientific evidence for precision medicine.

This study demonstrates several strengths in its design and data analysis strategy. Firstly, the study employed a rigorous cross-sectional design, ensuring the representativeness and reliability of the data. By precisely screening eligible patients with OSAS at a tertiary hospital in Zhejiang Province, the study effectively controlled for confounding factors by excluding patients with severe diseases, eye diseases, and mental disorders. This strict inclusion and exclusion criteria enhanced the internal validity of the study results. In terms of data collection, the study utilized ELISA technology to accurately measure levels of lactoferrin, lysozyme, and albumin in tear fluid, and polysomnography (PSG) was used to objectively assess the severity of OSAS. This multi-dimensional assessment approach enhanced the scientific rigor and accuracy of the study. The data analysis strategy is also commendable; the study systematically compared baseline characteristics and employed correlation and univariate analyses to comprehensively evaluate potential associations between dry eye biomarkers in tear and OSAS. Although the final results did not show statistical significance, the study’s rigor and systematic approach laid a crucial foundation for further in-depth research into the mechanisms of OSAS-related ocular complications.

This study has several limitations. As a single-center study in Zhejiang Province, its generalizability is limited. The study population is restricted to Chinese Han individuals, making it difficult to extrapolate to other ethnicities. The observational design can only reveal associations between tear biomarkers for dry eye and OSAS, without establishing causality. Additionally, the cross-sectional design prevents tracking dynamic changes in these factors. Future multi-center, longitudinal studies with larger sample sizes are needed to validate these findings.

## 5 Conclusion

This study investigates the link between tear lactoferrin, lysozyme, albumin levels and obstructive sleep apnea syndrome. There exists no significant correlation between dry eye tear biomarkers levels and disease severity in OSAS patients. Larger-scale studies are needed to validate these findings.

## Data Availability

The raw data supporting the conclusions of this article will be made available by the authors, without undue reservation.
